# Identification of ABCG2^+^ cells in nasopharyngeal carcinoma cells

**DOI:** 10.3892/or.2011.1618

**Published:** 2011-12-30

**Authors:** HONGBO ZHANG, WEIDONG LIU, XIANGLING FENG, LEI WANG, XINGJUN JIANG, DINGYANG LIU, LIHUA ZHANG, BIN ZHU, WEN ZHOU, WENTING JIA, GUIFEI LI, CAIPING REN

**Affiliations:** 1 Cancer Research Institute, Xiang-Ya School of Medicine, Central South University, Key Laboratory for Carcinogenesis of Chinese Ministry of Health, Key Laboratory for Carcinogenesis and Cancer Invasion of Chinese Ministry of Education, Hunan 410078; 2 Department of Neurosurgery, Xiangya Hospital, Central South University, Hunan 410008, P.R. China

**Keywords:** ABCG2, cancer stem cell, nasopharyngeal carcinoma

## Abstract

Tumor stem cells are a small subset of tumor cells with the ability of self-renewal and differentiation and are regarded as a cause of tumor growth and recurrence. Previously we have shown that stem-like label-retaining cells (LRCs) can be detected in nasopharynx, tongue, esophagus and xenograft tumors formed by nasopharyngeal carcinoma (NPC) cell lines (5–8F, 6–10B and TMNE). The present study aimed to identify ABCG2^+^ cells in 5–8F NPC cells and compare their tumorigenic potential with ABCG2^−^ cells, expecting that we can obtain insight into the mechanism of the differential phenotypes of ABCG2^+^ and ABCG2^−^ cells. By using magnetic cell sorting (MACS) method, we isolated ABCG2^+^ cells and ABCG2^−^ cells from 5–8F cells. Among these two subpopulations and unsorted 5–8F cells, the rate of ABCG2^+^ cells at G1 phase was highest, while the rate of ABCG2^−^ cells at S phase was highest, indicating that ABCG2^+^ cells were mostly quiescent. However, ABCG2^+^ cells showed lower cloning efficiency and tumorigenicity than ABCG2^−^ cells. We also used Affymetrix U133 plus 2.0 human whole genome expression chip to identify the gene expression profile of ABCG2^+^ and ABCG2^−^ cells and found that both subpopulations expressed some stem cell associated genes, e.g., PSCA, ABCG2 and ALPI were expressed in ABCG2^+^ cells, and K19, integrin α6, integrin β4, CD44 and K14 were expressed in ABCG2^−^ cells, suggesting there were stem cells in both ABCG2^+^ and ABCG2^−^ cells. Our data demonstrated that there exist ABCG2^+^ cells in NPC cells, but ABCG2 alone is not sufficient for isolating cancer stem cells in 5–8F NPC cells.

## Introduction

Stem cells, with the ability to proliferate infinitely through self-renewal and differentiation, can be isolated and cultured from inner cell mass of blastocyst (1), primordial germ cells (2), bone marrow (3), brain (4), skin (5), digestive canal (6), respiratory tract (7), cornea (8), muscle (9), liver (10), pancreas (11) and lung (12). Many tumors contain a sub-population of stem cells known as cancer stem cells (CSCs). CSC has unlimited potential for self-renewal and can drive tumorigenesis and develop multidrug resistance (13,14). To date, CSCs have been identified in human leukemia (15) and in solid tumors including breast (16), bladder (17), colorectal (18), gastric (19), hepatocellular (20) and lung carcinomas (21), malignant melanoma (22), nasopharyngeal (23), pancreatic (24), prostate (25) and renal carcinomas (26). However, the characterization of CSC remains insufficient and CSC has not been isolated from some tumors. CSC is regarded as the root of cancer, and thus should be more important for cancer therapy than other tumor cells. Therefore, CSC might be a good therapeutic target for cancer treatment.

Side population (SP) cells, originally isolated from murine hematopoietic stem cells using their characteristic to efflux Hoechst 33342 dye and FACS method (27), have been sorted from many normal human tissues such as heart (28), prostate (29), limbal epithelium (30), skin (31), mammary gland (32) and kidney (33), and have also isolated from human cancer cells such as small cell lung cancer (34), glioma (35), prostate cancer (36), leukemia (37), neuroblastoma (38), hepatoma (39), nasopharyngeal carcinoma (23), colorectal cancer (39), thyroid cancer (40) and lung cancer (41). Cancer SP cells exhibit stem cell-like functions such as resistance to chemotherapy drugs, clonogenic ability and tumorigenicity. Therefore, SP cells can be regarded as a kind of enriched CSCs.

The phenotype of SP cells depends on the expression of ABCG2, a member of ATP binding cassette (ABC) transporters which belong to one of the largest transmembrane protein families. They use ATP to transport various substrates across cell membranes. The substrates include chemotherapy drugs, metabolites and other compounds such as Hoechst 33342 dye. To present, about 50 ABC transporters have been identified (42) and are divided into seven subfamilies (from A to G), among which ABCG2 is the second member of the G subfamily. ABCG2 was first identified in doxorubicin-resistant human MCF-7 breast cancer cells and thus also named as breast cancer resistance protein (BCRP) (43). It is widely distributed in normal tissues and stem cells including SP cells. High expression of ABCG2 has been detected in CSCs isolated from embryonic cancer (44), retinoblastoma (45), lung (41), liver (46), pancreas (47) and gallbladder cancer (48).

Previously we have identified LRCs in nasopoharyngeal epithelia and NPC xenograft tissues with bromodeoxyuridine (BrdU) (49). In this study, we isolated ABCG2^+^ and ABCG2^−^ cells from 5–8F NPC cells by using MACS and then characterized their biological properties and expression profiles. Our results suggest that ABCG2 alone is insufficient to identify CSCs in 5–8F NPC cells.

## Materials and methods

### Ethics statement

All animal work was performed under the institutional guidelines approved by the Animal Care and Use Committee of Central South University. The present study was also approved ethically by the institutional review board of Central South University.

### Double labeling detection for LRC and ABCG2 expression in 5–8F cells

We used immunofluorescence method to detect LRCs and ABCG2 expression in 5–8F cells. Briefly, 5–8F cells was labeled with BrdU, inoculated into nude mice and traced for 8 weeks. Then, the tissue sections were made from formed tumor blocks, hydrated, treated with 3% H_2_O_2_ for 10 min to remove endogenous peroxidase, with 2 N hydrochloric acid for 30 min at 37°C, with 0.1 M sodium borate for 4 min and then treated with 0.25% trypsin and washed with PBS after each above treatment. The treated tissue sections was added with antibody for BrdU (Sigma, St. Louis, MO) at 4°C overnight. After washing with PBS, the sections was added with goat anti-mouse IgG-FITC antibody (Santa Cruz Biotechnology, Santa Cruz, CA) and incubated for 30 min at RT. After washing with PBS, the sections were blocked with normal goat serum, added with mouse anti-human ABCG2 antibody (BD Pharmingen, USA), for 30 min at RT, then added with Texas Red conjugated goat anti-mouse IgG (Santa Cruz Biotechology) and incubated for 30 min at RT. After washing with PBS, the sections were observed and photographed with a fluorescence microscope.

### Separation of ABCG2^+^ cells by MACS

5–8F NPC cells were harvested, prepared into single cell suspension and counted. Less than 10^8^ cells were obtained for cell sorting. The volume of cell suspension was adjusted to 200 μl with 1X PBS containing 0.5% bovine serum albumin (BSA). Mouse anti-human ABCG2 antibody (20 μl) was added to the cell solution, mixed and incubated for 20 min at 4–8°C. The cells were washed with 1X PBS containing 0.5% BSA three times and the cell volume was adjusted to 200 μl. The goat anti-mouse IgG2a bound with magnetic beads (Miltenyi Biotec, Germany) was added to the cell solution, mixed and incubated for 20 min at 4–8°C and then washed with 1X PBS containing 0.5% BSA. The cells were harvested and the volume of cell suspension was adjusted to 0.5 ml. The sorting column was fixed on magnetic sorting stand (Miltenyi Biotec) and equilibrated by applying 0.5 ml 1X PBS containing 0.5% BSA. The bound cell suspension (0.5 ml) was applied to the column and the effluent was harvested. 1X PBS (0.5 ml) containing 0.5% BSA was applied to the column two times. The effluent was ABCG2^−^ cell fraction. Another 1 ml of 1X PBS containing 0.5% BSA was applied to the column, the effluent was repeated for application to new columns and the ABCG2^+^ cells were subsequently enriched. The final effluent was centrifuged for 5 min at 4°C, 1,000 rpm to obtain the cells.

### Identification of sorting effect

We used immunocytochemistry method to identify the purity of ABCG2^+^ cells. Briefly, enriched ABCG2^+^ were made into cell pellets. The cell pellets were treated with 3% H_2_O_2_ for 10 min, with 0.25% trypsin for 15 min and blocked with normal goat serum for 20 min. The cells were added with mouse anti-human ABCG2 antibody and incubated at 4°C overnight. After washing with PBS, the cells were added with goat anti-mouse IgG-HRP and incubated for 30 min at RT. After washing with PBS, the cells were developed with AEC (Zhongshan Goldenbridge Biotech Co., Ltd., China), counterstained with hematoxylin and mounted with Glycerol vinyl alcohol aqueous mounting solution (GVA, Zymed Laboratories, Inc., USA). The red ABCG2^+^ cells were observed with an optical microscope and the purity was calculated.

We also used flow cytometry to identify the purity of ABCG2^+^ cells. The MACS-sorted ABCG2^+^ cells were resuspended, added with anti-human ABCG2 antibody-FITC (1:200), incubated for 30 min at 4–8°C and washed with 1 ml of 1X PBS containing 0.5% BSA three times. Then the purity of ABCG2^+^ cells was measured with flow cytometry (FACS Calibur, BD, USA). Unsorted 5–8F cells added with IgG-FITC were used as control.

### Cloning efficiency determination

Colony formation assay was performed as previously described (50). Briefly, single cell suspension was prepared from ABCG2^+^, ABCG2^−^ and unsorted 5–8F cells and counted. Each cell type was seeded in 12-well plates (200 cells/well) and cultured at 37°C for 14 days in an incubator with 5% CO_2_. Then the cells were fixed with methanol and stained with 0.4% crystal violet. Colonies containing at least 50 cells were counted under an inverse microscope. Cloning efficiency (%) = cell colony amounts/200 × 100%.

### Cell cycle analysis

ABCG2^+^, ABCG2^−^ and unsorted 5–8F cells (2×10^6^) were harvested, respectively, washed with PBS, fixed with 70% ice-cold ethanol for 30 min. The ethanol was discarded and the cells were resuspended in 500 μl PBS, added with RNase A to a final concentration of 100 μg/ml, incubated at 37°C for 30 min, stained with 20 μg/ml of propidium iodide (PI) for 30 min, measured with flow cytometry and analyzed with Mod Fit LT software.

### Analysis of tumorigenesis in NOD/SCID mice

ABCG2^+^, ABCG2^−^ and unsorted 5–8F cells (10^2^, 10^3^, 10^4^ and 10^5^ per each type) were injected s.c. into three 4–6 weeks old female NOD/SCID mice with body weight of 17–24 g (Shanghai Slac Laboratory Animal Co., Ltd., Shanghai, China), respectively. All the mice were sacrificed 6–16 weeks after injection and examined for tumors. The tumor blocks were dissected and made into tissue sections for inspection. Tumor blocks were dissected and fixed by immersion in 4% paraformaldehyde phosphate buffer. After fixation for 2–4 h, tissues were dehydrated, paraffin- embedded, sectioned at 4 μm and stained with haematoxylin and eosin (HE) for histological examination.

### Microarray analysis

Microarray analysis was performed as previously described (51). GeneChip Human Genome U133 Plus 2.0 was used to analyze the gene expression profile of ABCG2^+^ cells and ABCG2^−^ cells. The chip covers 47,400 transcripts and contains 38,500 known human genes. A probe hits only one genomic location; probes that can be mapped to the same target sequence in the correct direction are grouped together in the same probe set; each probe set consists of 10–20 pairs of 25 mer probes; each probe pair consists of two probe cells, one of which is perfect match and another of which is mismatch containing one base mismatch. The gene sequences are selected from GenBank, dbEST and RefSeq.

Total RNA of ABCG2^+^ cells and ABCG2^−^ cells was extracted and used to purify polyA^+^ mRNA. The cDNA, double strand DNA and biotin-labled cRNA were synthesized in turn. After fragmented, the labled cRNA was loaded on the gene chip for microarray analysis. Hybridization, elution and staining of the chip were conducted with Affymetrix Hybridization Oven 640 and Affymetrix Fluidics Station 450 according to the manufacturer’s instruction. After the chips were scanned, GCOS data processing software was used to calculate and process the obtained data. Before comparison of the results of two chips, the data of each chip were normalized to obtain reporter signal value. For screening the differentially expressed genes between ABCG2^+^ and ABCG2^−^ group, signal log ratio ≥1.0 or ≤-1.0 (indicating 2-fold upregulation or downregulation of gene expression level) was set as screening criterion.

### Verification of differentially expressed genes by RT-PCR

ABCG2^+^ and ABCG2^−^ cells were harvested, respectively. Total RNAs were extracted from the harvested cells with TRIzol reagent according to the manufacturer’s protocols and subsequently digested with DNase I to remove the residual amount of genomic DNA. RT-PCR was carried out with AMV reverse transcriptase system to detect the expression of selected genes. The PCR conditions were as follows: 3 min at 95°C; 40 sec at 94°C, 30 sec at 55–58°C and 50 sec at 72°C for appropriate cycles; 10 min at 72°C for extension. GAPDH was used as internal control. The used PCR primers and cycle number are shown in Table I. RT-PCR product bands were scanned with image analyzer (Pharmacia, USA) and the accumulated optical density value (IA) of each band was analyzed with Imagemaster VDS software.

### Geneontology analysis

GOSTAT (http://gostat.wehi.edu.au) was used to analyze and annotate the differentially expressed genes. GO provides three kinds of specifying terminology to describe the characteristics of gene products, including molecular function, biological process and cellular component.

### Statistical analysis

SPSS13.0 statistical software and one-way ANOVA were used to analyze the cloning efficiency data. GCOS was used to test gene expression level of ABCG2^+^ and ABCG2^−^ group and rank-test was applied to define the determinant interval. A P-value <0.05 was considered to be statistically significant.

## Results

### Double labeling detection of LRC and ABCG2 expression in 5–8F cells

**Figure 1:**
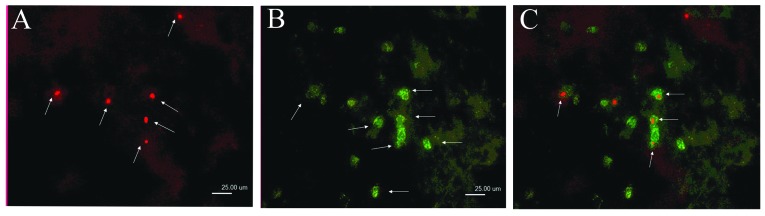
Detection of LRCs and ABCG2 expression in NPC tissues. (A) Detection of LRCs. Arrows indicate BrdU positive cells. (B) Detection of ABCG2 expression. Arrows indicate ABCG2 positive cells. (C) Double labeling for LRCs and ABCG2 expression. Arrows indicate double labeled cells. Red BrdU signals are concentrated in nuclei, while green ABCG2 signals are concentrated on cell membrane and in the cytoplasm.

5–8F NPC cells were labeled with BrdU, inoculated into nude mice, traced for 8 weeks and then detected for LRC and ABCG2 expression. In LRCs, there was 61.69±8.31% (n=3) of ABCG2^+^ cells, while in ABCG2^+^ cells, there was 12.05±2.80% (n=3) of LRCs (Table II, Fig. 1).

### Sorting ABCG2^+^ cells by MACS from 5–8F cells

**Figure 2:**
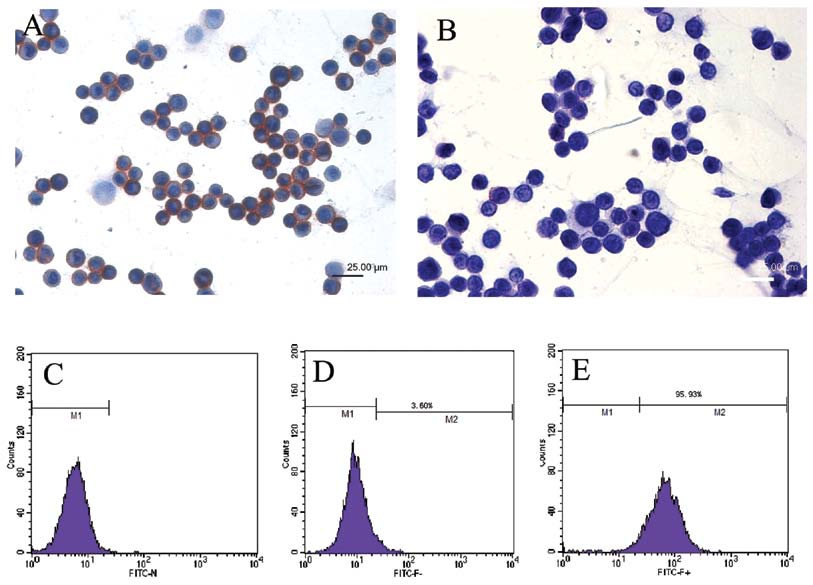
Verification of sorted cells by immunocytochemistry (A and B) and flow cytometry (C–E). ABCG2 is expressed only in ABCG2^+^ cells (A) but not in ABCG2^−^ cells (B). (C–E) Indicate measurement of ABCG2 expression by flow cytometry in 5–8F cells (C), ABCG2^−^ cells (D) and ABCG2^+^ cells (E), respectively. The purity of sorted ABCG2^+^ cells is 95.93%.

5–8F NPC cells were cultured and harvested, labeled with ABCG2 antibody and magnetic beads, and then sorted through MS sorting column. The rate of ABCG2^+^ cells was 2.11±0.36% (n=5). The ABCG2^+^ and ABCG2^−^ cells were smeared on slides, respectively and detected for ABCG2 expression with immunocytochemistry methods. ABCG2 was highly expressed in ABCG2^+^ cells, the positive signals located on the cell membrane and the purity of ABCG2^+^ cells reached 90.73%. ABCG2 was weakly expressed in only a minority of ABCG2^−^ cells (Fig. 2A and B). After labeled with IgG-FITC, ABCG2^+^ and ABCG2^−^ cells were analyzed by flow cytometry. It was shown that the purity of ABCG2^+^ cells was 95.93% (Fig. 2C–E). These data showed that we had successfully enriched ABCG2^+^ cells.

### Identification of biological characteristics of ABCG2^+^ cells

**Figure 3:**
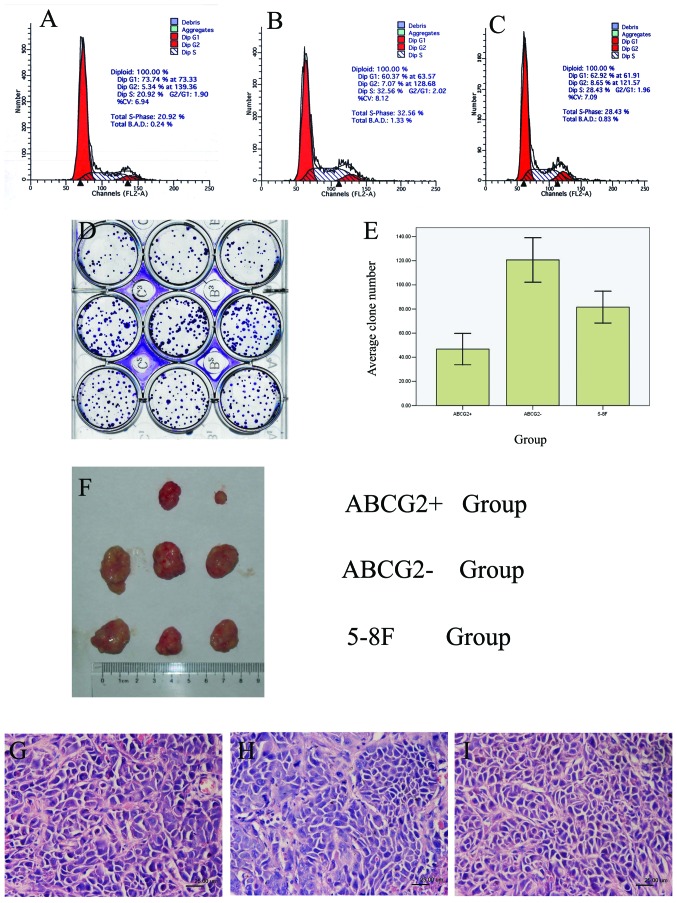
Biological characteristics of ABCG2^+^ cells. (A–C) The cell cycle distribution of ABCG2^+^, ABCG2^−^ and unsorted 5–8F cells, respectively. (D) Colonies formed by ABCG2^+^ cells (upper row), ABCG2^−^ cells (middle row) and 5–8F cells (lower row). Each kind of cells was inoculated into 3 wells at 200 cells per well. After 14 days, the colonies were stained with crystal violet and then observed and counted. (E) Colony formation rate with bar chart. (F) The tumor blocks formed by inoculation of ABCG2^+^, ABCG2^−^ and 5–8F cells into NOD/SCID mice. (G–I) H&E staining of tissue sections made from xenograft tumors formed by inoculation of ABCG2^+^ cells (G), ABCG2^−^ cells (H) and 5–8F cells (I) into NOD/SCID mice.

ABCG2^+^, ABCG2^−^ and unsorted 5–8F cells were analyzed by flow cytometry, respectively. As seen in Fig. 3A–C and Table III, the rate of G0/G1 phase cells was the highest (73.74%) in ABCG2^+^ cells among these three types of cells and the rate of S phase cells was the highest (32.56%) in ABCG2^−^ cells, indicating that ABCG2^+^ cells were mostly quiescent and more ABCG2^−^ cells were in DNA synthesis period. Therefore, some of the ABCG2^−^ cells might be the transient amplifying cells that could proliferate rapidly.

Cloning efficiency was analyzed among ABCG2^+^, ABCG2^−^ and unsorted 5–8F cells. The ABCG2^+^ cells formed smaller number of colonies compared with ABCG2^−^ cells and unsorted 5–8F cells (P<0.05), while the formed colony number of ABCG2^−^ cells was higher than that of 5–8F cells (P<0.05) (Fig. 3D and E, Table IV).

To compare the tumorigenicity of ABCG2^+^, ABCG2^−^ and unsorted 5–8F cells, we inoculated these cells into NOD/SCID mice, respectively. When inoculated with 10^2^–10^4^ cells, tumor formation could not be observed even after 113 days in either group. When inoculated with 10^5^ cells, tumor block could be seen after 12 days in ABCG2^−^ cell group and 5–8F cell group and after 20 days in ABCG2^+^ cell group. The weight of tumor blocks was highest in ABCG2^−^ cell group and lowest in ABCG2^+^ cell group (Fig. 3F, Table V). Tumor formation rate was lowest in ABCG2^+^ cell group (2/3) and was 100% (3/3) in other two groups. Paraffin sections were prepared from these tumor blocks and detected by H&E staining. The morphology of tumor cells from these three groups exhibited no significant difference (Fig. 3G–I).

### Gene expression profile of ABCG2^+^ and ABCG2^−^ cells

Affymetrix oligonucleotide microarray (Human Genome U133 Plus 2.0 Array) was used to monitor gene expression of about 47,400 transcripts containing 38,500 known genes in ABCG2^+^ and ABCG2^−^ cells. After the differential gene expression profiles between ABCG2^+^ and ABCG2^−^ cells were constructed, differentially expressed genes or ESTs which were upregulated (for 2 fold) or downregulated (for 2 fold) were screened. There were 353 genes or ESTs upregulated significantly in ABCG2^+^ cells and 590 genes or ESTs upregulated significantly in ABCG2^−^ cells out of the 47,400 transcripts. The 80 most significantly differentially expressed genes in ABCG2^+^ and ABCG2^−^ cells are listed in Table VI.

Differentially expressed genes were analyzed by Gene Ontology. A group of genes generally involved in negative regulation of cell cycle progression were discovered in ABCG2^+^ cells, whereas this functional classification could not be found in ABCG2^−^ cells, which can explain the fact that most ABCG2^+^ cells were in G0/G1 phase of cell cycle. The stem cell associated genes PSCA, ABCG2 and ALPI were upregulated significantly in ABCG2^+^ cells, while another set of stem cell related genes including K19, integrin α6, integrin β4, CD44 and K14 were upregulated significantly in ABCG2^−^ cells, suggesting that there are stem cells in both ABCG2^+^ and ABCG2^−^ cells.

**Figure 4:**
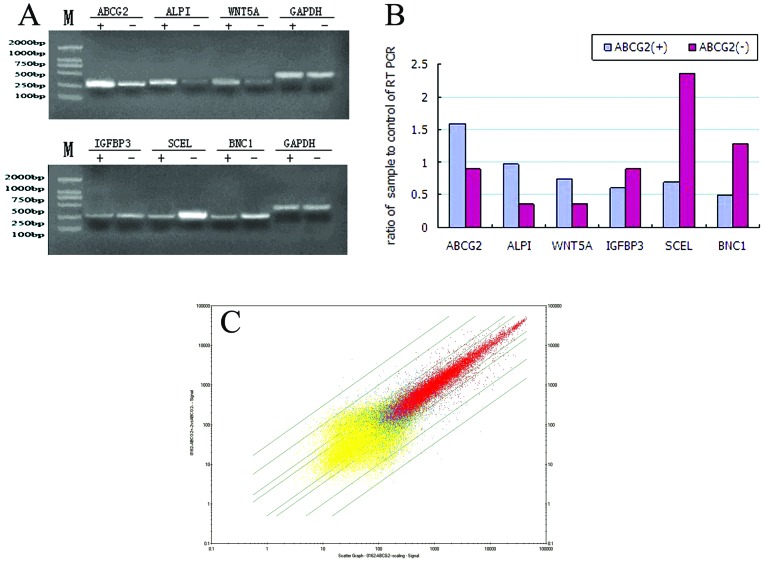
Gene expression profile of ABCG2^+^ and ABCG2^−^ cells. (A) Detection of differentially expressed genes by RT-PCR. ALPI, ABCG2 and WNT5A are highly expressed in ABCG2^+^ cells (upper figure), while IGFBP3, SCEL and BNC1 are highly expressed in ABCG2^−^ cells (lower figure). GAPDH is used as internal control. (B) Expression levels of detected genes shown with bar chart. (C) The scatterplot of average expression value in ABCG2^+^ and ABCG2^−^ cells.

From the chip analysis results, we selected 6 meaningful genes such as ALPI, ABCG2 and WNT5A which were highly expressed in ABCG2^+^ cells, and BNC1, IGFBP3 and SCEL which were highly expressed in ABCG2^−^ cells to perform RT-PCR verification. The expression of these genes was consistent with the results of chip analysis (Fig. 4A and B).

Fig. 4C exhibited the scatterplot of average expression value in ABCG2^+^ and ABCG2^−^ cells. The x-axis showed the signals of ABCG2^−^ cell group and the y-axis showed the signals of ABCG2^+^ cell group. Red plots indicated genes whose detection results were P (present) in two groups, blue blots indicated genes whose detection results were P in only one of the two groups and yellow plots represented genes whose detection results were A (absent) in both groups. From top to bottom, the green oblique lines represented expression difference at 30, 10, 4, 2, 1/2, 1/4, 1/10 and 1/30-fold between ABCG2^+^ and ABCG2^−^ cells, respectively. The result demonstrated that the expression difference of most genes between ABCG2^+^ and ABCG2^−^ cells was 2- to 4-fold and that only a minority of the genes could reach 10- to 30-fold or above.

## Discussion

Previously we have shown that LRCs exist in nasopharynx, tongue, esophagus and xenograft NPC tissues (49). One of the characteristics of adult stem cells is that they can be labeled for a long time and therefore are known as LRCs (52). Label retaining experiment is an effective method to label and detect stem cells in tissue of living organism (53). BrdU and 3H-thymine deoxyribose (3H-TdR) are commonly used labeling markers. The mechanism underlying the label retaining of a marker in stem cells is unclear. One explanation is that stem cells exhibit slow cell cycle progression, therefore the marker can remain in DNA of LRCs after tracing for a long time, while the marker in other cells will be gradually diluted with rapid cell division. Cairns (54) raised another explanation for LRC. Because of the asymmetry of stem cell division, labeled DNA is always allocated to daughter stem cells and the newly synthesized DNA is always allocated to daughter differentiated cells. We consider that the above explanations can partly explain the mechanism of the label retaining characteristic of stem cells.

In this study, we first labeled the cells in NPC tissue formed by inoculation of 5–8F cells into nude mice. Although scarce, the existence of LRCs indicated that there were cancer stem cells in NPC tissue. To further identify these LRCs, we detected the expression of ABCG2 in the same NPC tissue. ABCG2, a member of ABC transporter superfamily, is a transmembrane protein in charge of the efflux of chemotherapy drugs, metabolites and other compounds such as Hoechst 33342 dye, thus it is responsible for the phenotype of SP cells and has been widely used as a marker in CSCs isolated from retinoblastoma (45), embryonic (44), lung (41), liver (46), pancreas (47), gallbladder (48) and head and neck cancers (55) and NPC (23). Our results showed that there were approximately 62% of ABCG2^+^ cells in LRCs, suggesting that LRCs may represent a group of CSCs in NPC cells. This result is similar to that of research work of Welm *et al* (56). They found that the number of LRCs in SP cells was 4 times of those in non-SP cells.

Currently it is technically impossible to isolate LRCs from tumor tissue. We tried to sort ABCG2^+^ cells by MACS from 5–8F cells. The ABCG2-positive rate was 2.11%, which is similar to those in other tumor cells (57). Subsequently, we identified the biological characteristics of these ABCG2^+^ cells, and found that among ABCG2^+^, ABCG2^−^ and unsorted 5–8F cells, the rate of ABCG2^+^ cells were highest at G0/G1 phase, while the rate of ABCG2^−^ cells were highest at S phase, indicating that ABCG2^+^ cells were mostly quiescent, and more ABCG2^−^ cells were in DNA synthesis period. Therefore, some of the ABCG2^−^ cells might be the transient amplifying cells that could proliferate rapidly. Among the three kinds of cells, the cloning efficiency of ABCG2^+^ cells was lower than that of ABCG2^−^ cells and unsorted cells, and the tumorigenicity of ABCG2^+^ cells was also the lowest. We suppose that there may be several possibilities leading to the above results. One is that ABCG2 alone can not sufficiently enrich CSCs from 5–8F cells, therefore there are non-CSCs in ABCG2^+^ cells and there are some CSCs in ABCG2^−^ cells. Another is that ABCG2^+^ cells are enriched in SP cells, but are not equal to SP cells, thus may not exhibit typical properties of CSCs. Our results are similar to those of Patrawala *et al* (57). They found that side population isolated from prostate cancer, breast cancer and glioma was enriched in tumorigenic, stem-like cancer cells, whereas ABCG2^+^ and ABCG2^−^ cancer cells were similarly tumorigenic.

To explore the molecular mechanism underlying the biological characteristics of ABCG2^+^ cells, Affymetrix oligonucleotide microarray was used to monitor expression of about 47,400 transcripts containing 38,500 known genes in ABCG2^+^ and ABCG2^−^ cells. There were 353 genes and ESTs upregulated significantly and 590 genes downregulated significantly in ABCG2^+^ cells. As analyzed by Gene Ontology, a group of genes generally involving in negative regulation of cell cycle were discovered in ABCG2^+^ but not in ABCG2^−^ cells. The stem cell associated genes PSCA, ABCG2 and ALPI were upregulated significantly in ABCG2^+^ cells, while K19, integrin α6, integrin β4, CD44 and K14 were upregulated significantly in ABCG2^−^ cells. Together with the fact that the rate of LRC in ABCG2^+^ cells is only 12%, we suppose the most likely possibility is that ABCG2 alone is insufficient to mark CSCs in 5–8F cells. Further study waits to be conducted to isolate and identify CSCs from NPC cells and NPC tissue.

## Figures and Tables

**Table I:** Primers and cycle numbers used in RT-PCR analysis.

Primer name		Primer sequence from 5′ to 3′	PCR cycles
GAPDH	Sense:	5′-CCACCCATGGCAAATTCCATGGCA-3′	23
	Antisense:	5′-TCTAGACGGCAGGTCAGGTCCACC-3′	
ALPI	Sense:	5′-TTCCCATACCTGGCTCTGTC-3′	30
	Antisense:	5′-TGAGTACCAGTTGCGGTTCA-3′	
ABCG2	Sense:	5′-TGTGGAGGAACTGGGTAGGA-3′	28
	Antisense:	5′-AAGCCATTGGTGTTTCCTTG-3′	
WNT5A	Sense:	5′-CTCGCCATGAAGAAGTCCAT-3′	28
	Antisense:	5′-CCTTCGATGTCGGAATTGAT-3′	
BNC1	Sense:	5′-AACCCGGGAAAATAAACCAC-3′	30
	Antisense:	5′-ATGATGCACCAGTGATCCAA-3′	
IGFBP3	Sense:	5′-ACAGCCAGCGCTACAAAGTT-3′	30
	Antisense:	5′-AGGCTGCCCATACTTATCCA-3′	
SCEL	Sense:	5′-GTGGTGCTCAACCGACATAA-3′	32
	Antisense:	5′-TGCTCGAAGAGGCATTGTAA-3′	

**Table II:** Results of double labeling detection for LRCs and ABCG2 expression.

	DLCs/LRCs (%)	DLCs/ABCG2^+^ cells (%)
Xenografted nude mouse 1	60.12	9.64
Xenografted nude mouse 2	54.28	11.38
Xenografted nude mouse 3	70.67	15.12
Mean ± SD	61.69±8.31	12.05±2.80

DLCs, double labeled cells.

**Table III:** Cell cycle distribution of ABCG2^+^, ABCG2^−^ and unsorted 5–8F cells.

	Cell cycle distribution (%)		
	
Cells	G0+G1	S	G2+M
ABCG2^+^	73.74	20.92	5.34
ABCG2^−^	60.37	32.56	7.07
Unsorted 5–8F	62.92	28.43	8.65

**Table IV:** Comparison of colony-forming capacity of ABCG2^+^, ABCG2^−^ and unsorted 5–8F cells.

	ABCG2^+^	ABCG2^−^	5–8F
Colony no.	42, 48, 49	115, 129, 132	77, 86, 88
	46, 49, 57	104, 122, 129	69, 78, 82
	33, 48, 49	112, 118, 125	80, 83, 91
Mean ± SD^a^	46.78±6.48	120.67±9.22	81.56±6.58

a There is significant difference between the data of any two groups (P<0.05).

**Table V:** Tumors formed by dorsal subcutaneous inoculation of ABCG2^+^, ABCG2^−^ and unsorted 5–8F cells into NOD/SCID mice.

Cells	Cell no.	Tumor formation rate	Tumor weight (g)	Latency period (day)
ABCG2^+^	1×10^2^	-		
	1×10^3^	-		
	1×10^4^	-		
	1×10^5^	2/3	0.93, 0.17	20, 29
ABCG2^−^	1×10^2^	-		
	1×10^3^	-		
	1×10^4^	-		
	1×10^5^	3/3	2.44, 3.95, 2.29	12, 14, 14
Unsorted 5–8F	1×10^2^	-		
	1×10^3^	-		
	1×10^4^	-		
	1×10^5^	3/3	2.96, 1.43, 1.69	12, 13, 15

**Table VI:** Eighty most differentially expressed genes in ABCG2^+^ and ABCG2^−^ cells.

Gene symbol	SLR^a^	UniGene ID	Gene title	Chromosomal location
A2M	6.4	Hs.212838	α-2-macroglobulin	chr12p13.3-p12.3
ALPI	5.9	Hs.37009	Alkaline phosphatase, intestinal	chr2q37.1
CGA	5.7	Hs.119689	Glycoprotein hormones, α polypeptide	chr6q12-q21
SLC16A6	5.3	Hs.42645	Solute carrier family 16, member 6	chr17q24.2
DAB2	5.2	Hs.481980	Disabled homolog 2, mitogen-responsive phosphoprotein	chr5p13
C1QTNF6	4.9	Hs.22011	C1q and tumor necrosis factor related protein 6	chr22q13.1
SNAP25	4.6	Hs.167317	Synaptosomal-associated protein, 25 kDa	chr20p12-p11.2
DIO2	4.4	Hs.202354	Deiodinase, iodothyronine, type II	chr14q24.2-q24.3
CTGF	4.1	Hs.410037	Connective tissue growth factor	chr6q23.1
ECG2	4.1	Hs.244569	Esophagus cancer-related gene-2	chr5q32
PDE3A	4.1	Hs.386791	Phosphodiesterase 3A, cGMP-inhibited	chr12p12
TFPI	4.0	Hs.516578	Tissue factor pathway inhibitor (lipoprotein-associated coagulation inhibitor)	chr2q31-q32.1
ADAM12	4.0	Hs.386283	ADAM metallopeptidase domain 12 (meltrin α)	chr10q26.3
C6orf176	3.9	Hs.31917	Chromosome 6 open reading frame 176	chr6q27
EID3	3.9	-	E1A-like inhibitor of differentiation 3	chr12q23-q24.1
DUSP1	3.8	Hs.171695	Dual specificity phosphatase 1	chr5q34
PCSK1	3.6	Hs.78977	Proprotein convertase subtilisin/kexin type 1	chr5q15-q21
PRO0132	3.6	-	PRO0132 protein	chr2q34
SLC16A6	3.6	Hs.463838	Solute carrier family 16, member 6	chr17q24.2
MRS2L	3.4	Hs.533291	MRS2-like, magnesium homeostasis factor	chr6p22.3-p22.1
SLC29A3	3.3	Hs.438419	Solute carrier family 29, member 3	chr10q22.1
PRG1	3.2	Hs.1908	Proteoglycan 1, secretory granule	chr10q22.1
CES1	3.2	Hs.535486	Carboxylesterase 1	chr16q13-q22.1
UGT1A8	3.2	-	UDP glucuronosyltransferase 1 family, polypeptide A8	chr2q37
APOC3	3.1	Hs.534984	Apolipoprotein C-III	chr11q23.1-q23.2
ABCG2	3.1	Hs.480218	ATP-binding cassette, sub-family G, member 2	chr4q22
FYN	3.1	Hs.390567	FYN oncogene related to SRC, FGR, YES	chr6q21
WNT5A	3.1	Hs.152213	Wingless-type MMTV integration site family, member 5A	chr3p21-p14
FOSB	3.0	Hs.75678	FBJ murine osteosarcoma viral oncogene homolog B	chr19q13.32
PAPSS2	3.0	Hs.524491	3′-Phosphoadenosine 5′-phosphosulfate synthase 2	chr10q23-q24
VTN	3.0	Hs.2257	Vitronectin	chr17q11
CPS1	3.0	Hs.149252	Carbamoyl-phosphate synthetase 1	chr2q35
RHOBTB1	3.0	Hs.148670	Rho-related BTB domain containing 1	chr10q21.2
FTO	3.0	Hs.528833	Fatso	chr16q12.2
TBX3	3.0	Hs.129895	T-box 3	chr12q24.1
C20orf100	3.0	Hs.26608	Chromosome 20 open reading frame 100	chr20q13.12
BMP2	2.9	Hs.73853	Bone morphogenetic protein 2	chr20p12
PPP1R15A	2.8	Hs.76556	Protein phosphatase 1, regulatory (inhibitor) subunit 15A	chr19q13.2
MX1	2.8	Hs.517307	Myxovirus resistance 1	chr21q22.3
PAPSS2	2.8	Hs.524491	3′-Phosphoadenosine 5′-phosphosulfate synthase 2	chr10q23-q24
IF	−3.2	Hs.312485	I factor (complement)	chr4q25
TBX18	−3.2	Hs.251830	T-box 18	chr6q14-q15
ITGB4	−3.2	Hs.370255	Integrin, β 4	chr17q25
SLCO1B3	−3.2	Hs.504966	Solute carrier organic anion transporter family, member 1B3	chr12p12
CD300LG	−3.3	Hs.147313	CD300 antigen like family member G	chr17q21.31
GJA1	−3.3	Hs.74471	Gap junction protein, α 1, 43 kDa (connexin 43)	chr6q21-q23.2
FLI1	−3.4	Hs.504281	Friend leukemia virus integration 1	chr11q24.1-q24.3
IGFBP3	−3.4	Hs.450230	Insulin-like growth factor binding protein 3	chr7p13-p12
GABRB1	−3.4	Hs.27283	γ-aminobutyric acid (GABA) A receptor, β 1	chr4p12
LUM	−3.5	Hs.406475	Lumican	chr12q21.3-q22
CALB1	−3.5	Hs.65425	Calbindin 1, 28 kDa	chr8q21.3-q22.1
TP73L	−3.5	Hs.137569	Tumor protein p73-like	chr3q28
DSG3	−3.5	Hs.1925	Desmoglein 3 (pemphigus vulgaris antigen)	chr18q12.1-q12.2
PPP1R14C	−3.6	Hs.486798	Protein phosphatase 1, regulatory (inhibitor) subunit 14C	chr6q24.3-q25.3
OR5K1	−3.7	Hs.531371	Olfactory receptor, family 5, subfamily K, member 1	chr3q12.1
GPR87	−3.7	Hs.58561	G protein-coupled receptor 87	chr3q24
RSAD2	−3.7	Hs.17518	Radical S-adenosyl methionine domain containing 2	chr2p25.2
EGLN3	−3.8	Hs.135507	Egl nine homolog 3 (C. elegans)	chr14q13.1
CLCA2	−3.9	Hs.241551	Chloride channel, calcium-activated, family member 2	chr1p31-p22
FST	−3.9	Hs.9914	Follistatin	chr5q11.2
LOC196264	−4.0	Hs.15396	Hypothetical protein LOC196264	chr11q23.3
DSC3	−4.0	Hs.41690	Desmocollin 3	chr18q12.1
KRT14	−4.0	Hs.355214	Keratin 14 (epidermolysis bullosa simplex, Dowling-Meara, Koebner)	chr17q12-q21
PDCD8	−4.0	Hs.424932	Programmed cell death 8 (apoptosis-inducing factor)	chrXq25-q26
PRSS35	−4.0	Hs.98381	Protease, serine, 35	chr6q14.2
BNC1	−4.1	Hs.459153	Basonuclin 1	chr15q25.2
ITGB6	−4.1	Hs.470399	Integrin, β 6	chr2q24.2
SCEL	−4.1	Hs.492938	Sciellin	chr13q22
LAMC2	−4.2	Hs.530509	Laminin, γ 2	chr1q25-q31
PDZK3	−4.2	Hs.481819	PDZ domain containing 3	chr5p13.3
GABRA2	−4.3	Hs.116250	γ-aminobutyric acid (GABA) A receptor, α 2	chr4p12
LAMB4	−4.4	Hs.62022	Laminin, β 4	chr7q22-q31.2
KRT19	−4.5	Hs.514167	Keratin 19	chr17q21.2
DSG3	−4.5	Hs.1925	Desmoglein 3 (pemphigus vulgaris antigen)	chr18q12.1-q12.2
ITGB4	−4.6	Hs.370255	Integrin, β 4	chr17q25
ROCK1	−4.7	Hs.306307	Rho-associated, coiled-coil containing protein kinase 1	chr18q11.1
IGFBP7	−4.8	Hs.479808	Insulin-like growth factor binding protein 7	chr4q12
IGFBP3	−4.8	Hs.450230	Insulin-like growth factor binding protein 3	chr7p13-p12
LIFR	−4.8	Hs.133421	Leukemia inhibitory factor receptor	chr5p13-p12
IL1A	−5.5	Hs.1722	Interleukin 1, α	chr2q14

a SLR, ABCG2^+^ vs. ABCG2^−^ signal log ratio.
